# My sojourn with cerebral sympathetic nervous activity

**DOI:** 10.1113/EP092029

**Published:** 2024-07-20

**Authors:** Patrice Brassard

**Affiliations:** ^1^ Department of Kinesiology, Faculty of Medicine Université Laval Québec Canada; ^2^ Research center of the Institut universitaire de cardiologie et de pneumologie de Québec‐Université Laval Québec Canada

In 2006, I took one critical decision which would completely alter the trajectory of my career. Being in the final year of my PhD training under the mentorship of Dr Paul Poirier at Université Laval, Quebec, Canada, I decided The Copenhagen Muscle Research Center would be the place I wanted to complete my postdoctoral fellowship. Having focused my PhD work on exercise physiology in patients with type 2 diabetes (Brassard et al., 2006a, 2006b, 2007; Caron et al., 2017), I initially approached Prof. Michael Kjær at the annual meeting of the American College of Sports Medicine, which was in Denver, Colorado, in the same year. However, Prof. Kjær had recently changed his research focus away from diabetes, so it was not really possible for him to become my postdoc supervisor. During that brief meeting, he mentioned that one of his colleagues, who also happened to be present at the meeting, would most likely be interested in my profile. That colleague was Prof. Niels Secher (Aalkjaer et al., 2023). The rest is history!

During my stay in Copenhagen in Prof. Secher's laboratory, I had the opportunity to develop a new research expertise. While I examined the influence of well‐controlled type 2 diabetes on exercise responses during my PhD, I switched gears for my postdoc, and studied cerebral blood flow (CBF) regulation and cerebral metabolism in health and disease. For example, I got involved in a plethora of studies, looking at the impact of hypoxia (Avnstorp et al., 2015; Bailey et al., 2017; Overgaard et al., 2012), endotoxaemia (Brassard et al., 2012), and diabetes (Kim et al., 2015) on CBF and cerebral oxygenation ($S_{cO_{2}}$) at rest and during exercise. We also studied the impact of acute and chronic exercise on brain‐derived neurotrophic factor (Rasmussen et al., 2009; Seifert et al., 2010) and the influence of varying the speed of a left ventricular assist device during aerobic exercise on cerebral haemodynamics and exercise tolerance in patients in heart failure (Brassard et al., 2011). During that time period, I was introduced to the cerebral pressure–flow relationship and dynamic cerebral autoregulation concepts. I also learned the basics of transfer function analysis with Johannes van Lieshout (Brassard et al., 2012), a regular visiting scholar (and good friend) of Prof. Secher, who was present at the beginning of my stay in Denmark, ironically to start a study examining the influence of diabetes on CBF and $S_{cO_{2}}$ during aerobic exercise (Kim et al., 2015).

One clinical observation that intrigued us in regards to the cerebral pressure–flow relationship was that the administration of phenylephrine, an α_1_‐adrenergic agonist elevating mean arterial pressure (MAP) through total peripheral resistance, was associated with a reduction in $S_{cO_{2}}$. In a series of laboratory and clinical studies, we tried to better characterize the impact of phenylephrine, noradrenaline (NA; α‐adrenergic agonist) and ephedrine (α‐ and β‐adrenergic agonist) on $S_{cO_{2}}$ in healthy volunteers and patients (Brassard et al., 2009, 2010; Nissen et al., 2010). In one of these studies, we reported a reduction in near‐infrared spectroscopy‐derived $S_{cO_{2}}$, but an increase in middle cerebral artery mean blood velocity (MCAv_mean_), with administration of phenylephrine (Brassard et al., 2010). We speculated that these findings may have been the consequence of direct vasoconstrictive effects of phenylephrine on cerebral conduit and resistance vessels. An elegant and insightful invited editorial from Profs Philip Ainslie (University of British Columbia Okanagan, Canada) and Yu‐Chieh Tzeng (University of Otago, New Zealand) questioned whether these findings were really explained by the direct action of phenylephrine per se, or rather changes occurring secondary to the transient increase in MAP (Ainslie & Tzeng, 2010). They also mentioned: ‘Therefore, the findings of Brassard et al. call for new research to determine if there is differential regulation of peripheral vs. cerebral sympathetic nerve activity in humans but, importantly, to explain how cerebral sympathetic activity contributes to CBF control, given the apparently low innervation density of human cerebral resistance vessels’ (Ainslie & Tzeng, 2010).

This stimulating scientific exchange with leaders in the field ignited my ongoing interest for the role of (cerebral) sympathetic nervous activity (SNA) in CBF regulation. Soon after the publication of this paper and its accompanying editorial, Prof. Secher and I started to discuss the possibility of examining this question in his laboratory (note that I had completed my postdoc at that time, being an assistant professor in the Division of Kinesiology [which became the Department of Kinesiology in 2012] at Université Laval since 2009). In 2013, I received an invitation from Prof. Ainslie to co‐write a review paper on that specific topic. Studying the role of cerebral SNA on CBF in humans remains a difficult task. The human cerebral circulation is richly innervated with sympathetic nerve fibres, but whether SNA has an influence on CBF and cerebral autoregulation was controversial at the time (and even today!) (Strandgaard & Sigurdsson, 2008; ter Laan et al., 2013; van Lieshout & Secher, 2008). In this review paper, we highlighted the possible reasons behind the controversy of the sympathetic control of cerebral autoregulation (Ainslie & Brassard, 2014). This article has attracted some attention and a decent number of citations since its first appearance online (128 citations according to Google Scholar as of June 2024).

Several reasons may be responsible for the contradictory conclusions reported in the literature in regards to the sympathetic control of the brain circulation, such as the presence of redundant mechanisms within the brain, heterogeneous distribution of sympathetic innervation, the influence of the experimental model used on the blood–brain barrier permeability, species differences in cerebrovascular responsiveness to sympathetic nerve stimulation, variation in the duration and the intensity of sympathetic stimulation, the influence of perfusion pressure, the various assessment methodologies used for CBF quantification and experimental approaches that can or cannot provide insight into the sympathetic control of CBF (reviewed in Ainslie & Brassard, 2014; Brassard et al., 2017). In 2016, Prof. Ainslie, Prof. Michael Tymko (a PhD student in Prof. Ainslie's laboratory at that time), and I received an invitation from Prof. Kevin Shoemaker, who was organizing a special issue for the journal *Autonomic Neuroscience* entitled ‘Imaging in Autonomic Neuroscience: Seeing is believing’, to submit a review article focusing on the sympathetic control of the cerebral circulation. In this review article, we provided an overview of the role of neural control in the regulation of CBF, with a focus on SNA, and discussed the above‐mentioned reasons behind the controversial influence of SNA on CBF regulation. We also critically reviewed the diverse methods of measuring CBF (Brassard et al., 2017).

Speaking of methods, microneurography and plasma NA are commonly used experimental techniques to evaluate SNA. However, whether peripheral SNA accurately reflects cerebral SNA in humans at rest, or during physiological stresses, remains debated. One promising approach to quantify cerebral SNA in humans is the brain NA spillover method. This powerful technique, developed by Prof. Murray Esler, from the Baker IDI Heart and Diabetes Institute in Australia, represents a neurochemical method to estimate total and regional SNA. Interestingly, Prof. Secher had previously utilized this technique with Prof. Esler (Mitchell et al., 2009). But as previously mentioned, I now run my own research laboratory in Quebec, Canada, far from Copenhagen to complete such invasive studies. This is the moment where I started an interesting journey to implement the brain NA spillover technique, and complete the first study using this method, in my research laboratory, which culminated in a first publication in 2024. One thing is crystal clear, this 9‐year path towards publication using the brain NA spillover method has been far from a long quiet river!

The first logical step to begin with was to approach Prof. Esler. After a successful first contact, Prof. Esler and I exchanged emails on a regular basis during the following months for us to better understand the technique and how to prepare tritiated NA infusion. Then there came Ms. Nathalie Châteauvert, another critical person for the implementation of this technique in my laboratory. Pharmacist and coordinator of the research pharmacy at the *Institut universitaire de cardiologie et de pneumologie de Québec‐Université Laval*, she was responsible for developing the process of preparing the tritiated NA tracer to be administered continuously to participants. Considering the pharmacy at our institution was not equipped to prepare this type of tracer, Nathalie Châteauvert took the necessary steps to secure the collaboration with our nuclear medicine department. In other words, she stepped up to the plate to complete this essential step of the study.

Then, we had to find an experienced clinician to insert catheters, especially the one placed retrograde in the internal jugular vein, to get simultaneous venous and arterial samples across the brain (essential for the brain NA spillover technique). While this procedure was routine in Prof. Secher's laboratory, clinicians from our institution with such experience could be counted on the fingers of one hand. Luckily, a common acquaintance mentioned to me, several months before the beginning of the study, that Dr Stephan Langevin, an anaesthesiologist at our institution who were treating patients with intracranial pressure, had experience with inserting catheters retrograde in internal jugular veins. Importantly, he was very keen on collaborating with us on this study. Good news! We now had our clinician to insert catheters. Since this procedure could not be physically completed in my laboratory, we had to collaborate with other clinicians to access an operating room for the completion of this key task. Dr Marc Fortin, a pneumologist at our institution, gave us access to the invasive bronchoscopy room he was regularly using, including all the necessary equipment and, importantly, all his staff available before their work shift, for catheter insertion.

A few years ago, my laboratory did not have all the necessary equipment to complete such a complex study. Prof. Ainslie was already aware that I was planning to complete this invasive study back in 2015, since I had asked him to review my grant application focusing on the role of cerebral SNA on CBF regulation in response to transient increases in arterial blood pressure (on a side note, it took me 5 years to get funding for this research programme!). Without any hesitation, he sent several pieces of equipment to my laboratory for the whole duration of the study. In addition, two of his PhD students, Michael Tymko and Geoff Coombs, were available and interested in contributing to the study. All this at no cost. Needless to say that this brain NA spillover study would not have been possible without Prof. Ainslie. The research team was completed by Julie Desjardins and Vickie Michaud, two skilled nurses, as well as my graduate students at the time (Audrey Drapeau, Lawrence Labrecque, Sarah Imhoff and Kevan Rahimaly) and a group of undergraduate students. In May and June 2018, 12 participants successfully completed the study in 9 days! An important achievement for the research team. But the story was far from being over…

For instance, the NA analysis ended up being far more complicated than initially planned. Before the beginning of the study, I had secured a collaboration with a colleague at my institution who would take care of tritiated and non‐tritiated NA analysis. Unfortunately, for several methodological reasons, those analyses could not be accomplished. Following 9 years of planning and the successful completion of this first brain NA spillover study, I became afraid we could not publish our findings because of difficulties analysing NA. Over the following months, I desperately searched for collaborators to complete the NA analyses. Disappointed and a bit discouraged, I did something I am totally against: a ‘Reply to all’. In 2020, I received an invitation to contribute to a special issue dedicated to the autonomic nervous system and cerebral autoregulation. Specifically, the special issue of this journal was devoted to the exploration of the link between autonomic function and cerebral autoregulation via diverse methodological approaches and experimental procedures. Without hesitation, I utilized the ‘Reply to all’ option to take advantage of this invite (sent to several SNA specialists) to submit my request to a group of experts in the SNA field. My request was very simple: ‘I was wondering whether one of you (or one of your colleagues) has expertise with plasma and tritiated NA analysis? I am actively looking for a collaborator.’ Just a few hours later, Dr Pedro Castro, a stroke neurologist and professor at the Faculty of Medicine, University of Porto, Portugal, wrote back mentioning that a colleague of his, Prof. Maria Augusta Vieira‐Coelho, a top leader in that specific research field with a vast experience and several publications with catecholamine measurements in plasma, was interested in contributing to our study. This is how we finally found the best expert available to complete our non‐tritiated and tritiated NA analysis.

The route was now clear and publication was finally within reach! Well, not so fast. Initially, a few plasma samples were shipped to Prof. Vieira‐Coelho to ensure the analyses could be completed. After having received confirmation that everything was working, I then decided to ship all samples to Portugal (it was actually half of all aliquots since we managed to get several aliquots for each blood sample). Without any experience with blood sample shipment, I was naïve enough to use a non‐specialized shipping company. What could go wrong? Well, our samples arrived in slush in Prof. Vieira‐Coelho's laboratory more than 2 weeks after departure. Half of our blood samples destroyed because of a novice error! Luckily, the other half of our samples were still sitting in our −80°C freezer. Lesson learned! I contacted a specialized company and the blood sample shipment was successfully and rapidly completed this time.

In April 2024, we finally published the first paper related to this ambitious study, examining the impact of aerobic and isometric exercise on cerebral SNA in healthy humans (Tymko et al., 2024). A second manuscript is presently under review and the remaining blood samples have been shipped to Prof. Damian Bailey at the University of South Wales, UK, to address a different research question related to cerebral SNA. The 9‐year period preceding the first publication related to our brain NA spillover study has been a roller coaster, associated with a lot of discussion, planning, troubleshooting, disappointment, excitement and pride. We are now all ready to tackle the next chapter of this cerebral SNA story!

## AUTHOR CONTRIBUTIONS

Sole author.

## CONFLICT OF INTEREST

The author declares he has no conflicts of interest.

## FUNDING INFORMATION

No funding was received for this work.

## References

[eph13607-bib-0001] Aalkjaer, C. , Fischer, M. , & Bailey, D. M. (2023). Professor Niels Henry Secher: Celebrating success from boat to bench to bedside. Experimental Physiology, 108(10), 1233–1234. 37647131 10.1113/EP091460 PMC10988488

[eph13607-bib-0002] Ainslie, P. N. , & Brassard, P. (2014). Why is the neural control of cerebral autoregulation so controversial? F1000 Medicine Reports, 6, 14. 10.12703/P6-14 PMC3944747 24669295

[eph13607-bib-0003] Ainslie, P. N. , & Tzeng, Y. C. (2010). On the regulation of the blood supply to the brain: Old age concepts and new age ideas. Journal of Applied Physiology, 108(6), 1447–1449. 20299620 10.1152/japplphysiol.00257.2010

[eph13607-bib-0004] Avnstorp, M. B. , Rasmussen, P. , Brassard, P. , Seifert, T. , Overgaard, M. , Krustrup, P. , Secher, N. H. , & Nordsborg, N. B. (2015). Cerebral water and ion balance remains stable when humans are exposed to acute hypoxic exercise. High Altitude Medicine & Biology, 16(1), 18–25. 25761236 10.1089/ham.2014.1075

[eph13607-bib-0005] Bailey, D. M. , Rasmussen, P. , Overgaard, M. , Evans, K. A. , Bohm, A. M. , Seifert, T. , Brassard, P. , Zaar, M. , Nielsen, H. B. , Raven, P. B. , & Secher, N. H. (2017). Nitrite and S‐nitrosohemoglobin exchange across the human cerebral and femoral circulation: Relationship to basal and exercise blood flow responses to hypoxia. Circulation, 135(2), 166–176. 27881556 10.1161/CIRCULATIONAHA.116.024226

[eph13607-bib-0006] Brassard, P. , Ferland, A. , Bogaty, P. , Desmeules, M. , Jobin, J. , & Poirier, P. (2006a). Influence of glycemic control on pulmonary function and heart rate in response to exercise in subjects with type 2 diabetes mellitus. Metabolism, 55(11), 1532–1537. 17046557 10.1016/j.metabol.2006.06.025

[eph13607-bib-0007] Brassard, P. , Ferland, A. , Gaudreault, V. , Bonneville, N. , Jobin, J. , & Poirier, P. (2006b). Elevated peak exercise systolic blood pressure is not associated with reduced exercise capacity in subjects with Type 2 diabetes. Journal of Applied Physiology, 101(6), 893–897. 16728521 10.1152/japplphysiol.00260.2006

[eph13607-bib-0008] Brassard, P. , Jensen, A. S. , Nordsborg, N. , Gustafsson, F. , Moller, J. E. , Hassager, C. , Boesgaard, S. , Hansen, P. B. , Olsen, P. S. , Sander, K. , Secher, N. H. , & Madsen, P. L. (2011). Central and peripheral blood flow during exercise with a continuous‐flow left ventricular assist device: Constant versus increasing pump speed: A pilot study. Circulation: Heart Failure, 4(5), 554–560. 21765126 10.1161/CIRCHEARTFAILURE.110.958041

[eph13607-bib-0009] Brassard, P. , Kim, Y. S. , van Lieshout, J. , Secher, N. H. , & Rosenmeier, J. B. (2012). Endotoxemia reduces cerebral perfusion but enhances dynamic cerebrovascular autoregulation at reduced arterial carbon dioxide tension. Critical Care Medicine, 40(6), 1873–1878. 22610190 10.1097/CCM.0b013e3182474ca7

[eph13607-bib-0010] Brassard, P. , Legault, S. , Garneau, C. , Bogaty, P. , Dumesnil, J. G. , & Poirier, P. (2007). Normalization of diastolic dysfunction in type 2 diabetics after exercise training. Medicine and Science in Sports and Exercise, 39(11), 1896–1901. 17986895 10.1249/mss.0b013e318145b642

[eph13607-bib-0011] Brassard, P. , Seifert, T. , & Secher, N. H. (2009). Is cerebral oxygenation negatively affected by infusion of norepinephrine in healthy subjects? British Journal of Anaesthesia, 102(6), 800–805. 19376788 10.1093/bja/aep065

[eph13607-bib-0012] Brassard, P. , Seifert, T. , Wissenberg, M. , Jensen, P. M. , Hansen, C. K. , & Secher, N. H. (2010). Phenylephrine decreases frontal lobe oxygenation at rest but not during moderately intense exercise. Journal of Applied Physiology, 108(6), 1472–1478. 20223999 10.1152/japplphysiol.01206.2009

[eph13607-bib-0013] Brassard, P. , Tymko, M. M. , & Ainslie, P. N. (2017). Sympathetic control of the brain circulation: Appreciating the complexities to better understand the controversy. Autonomic Neuroscience, 207, 37–47. 28506501 10.1016/j.autneu.2017.05.003

[eph13607-bib-0014] Caron, J. , duManoir, G. R. , Labrecque, L. , Chouinard, A. , Ferland, A. , Poirier, P. , Legault, S. , & Brassard, P. (2017). Impact of type 2 diabetes on cardiorespiratory function and exercise performance. Physiological Reports, 5(4), e13145. 28242825 10.14814/phy2.13145 PMC5328776

[eph13607-bib-0015] Kim, Y. S. , Seifert, T. , Brassard, P. , Rasmussen, P. , Vaag, A. , Nielsen, H. B. , Secher, N. H. , & van Lieshout, J. J. (2015). Impaired cerebral blood flow and oxygenation during exercise in type 2 diabetic patients. Physiological Reports, 3(6), e12430. 26109188 10.14814/phy2.12430 PMC4510631

[eph13607-bib-0016] Mitchell, D. A. , Lambert, G. , Secher, N. H. , Raven, P. B. , van Lieshout, J. , & Esler, M. D. (2009). Jugular venous overflow of noradrenaline from the brain: A neurochemical indicator of cerebrovascular sympathetic nerve activity in humans. The Journal of Physiology, 587(Pt 11), 2589–2597. 19403604 10.1113/jphysiol.2008.167999 PMC2714023

[eph13607-bib-0017] Nissen, P. , Brassard, P. , Jorgensen, T. B. , & Secher, N. H. (2010). Phenylephrine but not ephedrine reduces frontal lobe oxygenation following anesthesia‐induced hypotension. Neurocritical Care, 12(1), 17–23. 19957053 10.1007/s12028-009-9313-x

[eph13607-bib-0018] Overgaard, M. , Rasmussen, P. , Bohm, A. M. , Seifert, T. , Brassard, P. , Zaar, M. , Homann, P. , Evans, K. A. , Nielsen, H. B. , & Secher, N. H. (2012). Hypoxia and exercise provoke both lactate release and lactate oxidation by the human brain. Federation of American Societies for Experimental Biology Journal, 26(7), 3012–3020. 22441982 10.1096/fj.11-191999

[eph13607-bib-0019] Rasmussen, P. , Brassard, P. , Adser, H. , Pedersen, M. V. , Leick, L. , Hart, E. , Secher, N. H. , Pedersen, B. K. , & Pilegaard, H. (2009). Evidence for a release of brain‐derived neurotrophic factor from the brain during exercise. Experimental Physiology, 94(10), 1062–1069. 19666694 10.1113/expphysiol.2009.048512

[eph13607-bib-0020] Seifert, T. , Brassard, P. , Wissenberg, M. , Rasmussen, P. , Nordby, P. , Stallknecht, B. , Adser, H. , Jakobsen, A. H. , Pilegaard, H. , Nielsen, H. B. , & Secher, N. H. (2010). Endurance training enhances BDNF release from the human brain. American Journal of Physiology‐Regulatory, Integrative and Comparative Physiology, 298(2), R372–R377. 19923361 10.1152/ajpregu.00525.2009

[eph13607-bib-0021] Strandgaard, S. , & Sigurdsson, S. T. (2008). Point: Counterpoint: Sympathetic activity does/does not influence cerebral blood flow. Counterpoint: Sympathetic nerve activity does not influence cerebral blood flow. Journal of Applied Physiology, 105(4), 1366–1367. discussion 1367–1368. 18838509 10.1152/japplphysiol.90597.2008a

[eph13607-bib-0022] ter Laan, M. , van Dijk, J. M. C. , Elting, J. W. J. , Staal, M. J. , & Absalom, A. R. (2013). Sympathetic regulation of cerebral blood flow in humans: A review. British Journal of Anaesthesia, 111(3), 361–367. 23616589 10.1093/bja/aet122

[eph13607-bib-0023] Tymko, M. M. , Drapeau, A. , Vieira‐Coelho, M. A. , Labrecque, L. , Imhoff, S. , Coombs, G. B. , Langevin, S. , Fortin, M. , Chateauvert, N. , Ainslie, P. N. , & Brassard, P. (2024). Acute isometric and dynamic exercise do not alter cerebral sympathetic nerve activity in healthy humans. Journal of Cerebral Blood Flow and Metabolism. Advance online publication. 10.1177/0271678X241248228 PMC11639215 38613232

[eph13607-bib-0024] van Lieshout, J. J. , & Secher, N. H. (2008). Point:Counterpoint: Sympathetic activity does/does not influence cerebral blood flow. Point: Sympathetic activity does influence cerebral blood flow. Journal of Applied Physiology, 105(4), 1364–1366. 18583376 10.1152/japplphysiol.90597.2008

